# Leveraging Image Analysis to Compute 3D Plant Phenotypes Based on Voxel-Grid Plant Reconstruction

**DOI:** 10.3389/fpls.2020.521431

**Published:** 2020-12-09

**Authors:** Sruti Das Choudhury, Srikanth Maturu, Ashok Samal, Vincent Stoerger, Tala Awada

**Affiliations:** ^1^School of Natural Resources, University of Nebraska-Lincoln, Lincoln, NE, United States; ^2^Department of Computer Science and Engineering, University of Nebraska-Lincoln, Lincoln, NE, United States; ^3^Agricultural Research Division, University of Nebraska-Lincoln, Lincoln, NE, United States

**Keywords:** 3D plant voxel-grid reconstruction, 3D plant phenotyping taxonomy, Plant component separation, 3D phenotype computation, benchmark dataset

## Abstract

High throughput image-based plant phenotyping facilitates the extraction of morphological and biophysical traits of a large number of plants non-invasively in a relatively short time. It facilitates the computation of advanced phenotypes by considering the plant as a single object (holistic phenotypes) or its components, i.e., leaves and the stem (component phenotypes). The architectural complexity of plants increases over time due to variations in self-occlusions and phyllotaxy, i.e., arrangements of leaves around the stem. One of the central challenges to computing phenotypes from 2-dimensional (2D) single view images of plants, especially at the advanced vegetative stage in presence of self-occluding leaves, is that the information captured in 2D images is incomplete, and hence, the computed phenotypes are inaccurate. We introduce a novel algorithm to compute 3-dimensional (3D) plant phenotypes from multiview images using voxel-grid reconstruction of the plant (3DPhenoMV). The paper also presents a novel method to reliably detect and separate the individual leaves and the stem from the 3D voxel-grid of the plant using voxel overlapping consistency check and point cloud clustering techniques. To evaluate the performance of the proposed algorithm, we introduce the University of Nebraska-Lincoln 3D Plant Phenotyping Dataset (UNL-3DPPD). A generic taxonomy of 3D image-based plant phenotypes are also presented to promote 3D plant phenotyping research. A subset of these phenotypes are computed using computer vision algorithms with discussion of their significance in the context of plant science. The central contributions of the paper are (a) an algorithm for 3D voxel-grid reconstruction of maize plants at the advanced vegetative stages using images from multiple 2D views; (b) a generic taxonomy of 3D image-based plant phenotypes and a public benchmark dataset, i.e., UNL-3DPPD, to promote the development of 3D image-based plant phenotyping research; and (c) novel voxel overlapping consistency check and point cloud clustering techniques to detect and isolate individual leaves and stem of the maize plants to compute the component phenotypes. Detailed experimental analyses demonstrate the efficacy of the proposed method, and also show the potential of 3D phenotypes to explain the morphological characteristics of plants regulated by genetic and environmental interactions.

## 1. Introduction

The complex interaction between a genotype and its environment determines the observable phenotypic characteristics of a plant that influence resource acquisition and yield (Das Choudhury et al., 2019). High throughput image-based plant phenotyping refers to the non-invasive monitoring and quantification of plants' morphological and biophysical traits by analyzing their images captured at regular intervals with precision (Das Choudhury et al., 2018, 2019). It facilitates the analysis of a large number of plants in a relatively short time with no or little manual intervention to compute diverse phenotypes. The process is generally non-destructive, allowing the same traits to be quantified repeatedly at multiple times during a plant's life cycle. Extracting meaningful numerical phenotypes based on image analysis remains a critical bottleneck in plant science. Current image-based plant phenotyping methods have mainly focused on the computation of phenotypes, e.g., morphological, architectural, textural and color-based, from 2D plant image sequences for vegetative and reproductive stages (Dellen et al., 2015; Brichet et al., 2017; Das Choudhury et al., 2017, 2018; Pound et al., 2017; Zhang et al., 2017; Yin et al., 2018). However, plants, 3D in nature, exhibit increasing architectural complexity over time due to self-occlusions and phyllotaxy (i.e., arrangements of leaves around the stem), which pose significant challenges in the attempt to accurately estimating phenotypes from a 2D image and linking these phenotypes to genetic expression. For example, the stem angle determination method in Das Choudhury et al. (2017) from 2D single view image sequences (at which the line of sight of the camera is perpendicular to the line of axis of the leaves) in the 2D space, is unable to account for stem lodging toward and away from the camera. Furthermore, accurate estimation of the 3D structure of a plant to compute 3D phenotypes is important for the study of physiological processes in plants, e.g., plant leaf area and leaf angle significantly influence light interception, and thereby, transpiration, photosynthesis, and plant productivity (Thapa et al., 2018).

The method by Bosquet et al. (2016) experimentally demonstrated the temporal variation of leaf angle and leaf area induced by light interception based on 3D reconstruction of maize plants from multiple 2D side view images. The method by McCormick et al. (2016) used a depth camera for 3D reconstruction of the sorghum plants, and identified quantitative trait loci regulating sorghum architecture for the measurements of shoot height, leaf angle, leaf length, and shoot compactness. The method by Golbach et al. (2016) used the shape-from-silhouette method for reconstructing a 3D model of a tomato seedling based on images captured from ten calibrated cameras. The individual components of the plants, i.e., stem and leaves, were segmented from the 3D model to determine stem height and area of each leaf. A low-cost multiview stereo imaging system was described in He et al. (2017) for 3D point cloud reconstruction of strawberry fruits. The model was used to estimate seven agronomically important strawberry traits, i.e., height, length, width, volume, calyx size, color, and achene number. The method in Srivastava et al. (2017) described an algorithm for 3D model reconstruction of wheat plants with occluded leaves for drought stress characterization using deep learning techniques. A depth camera was used to acquire images of sorghum plants, and a semi-automated software pipeline was developed to generate 3D plant reconstructions from the images, which were used to compute standard measures of shoot architecture such as shoot height, leaf angle, leaf length and shoot compactness (McCormick et al., 2016).

Most of the existing methods (Klodt and Cremers, 2015; Golbach et al., 2016; Scharr et al., 2017) have focused on the 3D reconstruction of seedlings or early growth stages. Note that the smaller plants are characterized by architectural simplicity due to the absence of self-occlusions and concavities, and hence, their reconstructions are easier and less error prone. Unlike the previous studies, we aim to reconstruct plants at later vegetative stages to address the complexity that arise from self-occlusions and leaf crossovers. McCormick et al. (2016) performed 3D reconstruction of sorghum plants using depth images to identify quantitative trait loci for characterizing shoot architecture. However, the procedure required manual transportation of the plants from the greenhouse to a turn-table for imaging that resulted in a low throughput analysis. A fast high resolution volume carving method using octree was presented by Scharr et al. (2017) for shoot reconstruction of maize and banana seedlings. The method performed reconstruction using five maize plants from seedling to 2–8 leaves stage. The images were captured in a semi-automated system that required manual positioning of the plant on a turntable. The banana seedlings were imaged in an automatic screenhouse system, however, the plant height was limited to few centimeters. Furthermore, the method computed only three well-known phenotypic traits, i.e., the volume of visual hull, leaf-count and area of each leaf. The method in Guan et al. (2018) used a density-based spatial clustering algorithm for 3D reconstruction of soybean canopies to compute phenotypes. A multi-source imaging system consisting of a photonic mixer detector and a RGB camera was used to capture images of soybean plants placed at a distance of 80 cm to capture multiview images in an outdoor environment. The method in Wu et al. (2019) used Laplacian skeleton extraction to extract the skeleton of the 3D point cloud of a maize plant. It used color information to estimate phenotypes, e.g., leaf length, leaf inclination angle, leaf top length, leaf azimuthal angle, leaf growth height, and plant height. The method attempted to analyze plants after silking stage with reported limitations of unsatisfactory skeletonization of upper plant part where leaves were incompletely unfolded and also the reproductive organs, i.e., tassel and ear. The 3D point clouds were obtained using a terrestrial laser scanner (Faro Focus3D X130) in a low throughput indoor setting, where six plants were scanned at a time.

In this paper, we present a novel method called 3DPhenoMV for computing 3D plant phenotypes based on a voxel-grid reconstruction approach using multiview visible light image sequences captured in an automated high throughput plant phenotyping platform (HTP3) where the distance between the pot and the camera is significantly larger (5.5 m) compared to the state-of-the methods. 3DPhenoMV uses a well-known space carving technique for voxel-grid reconstruction and aims to achieve the fully automatic reconstruction of a large number of plants at their late vegetative stages (with plant's height up to 2.5 m) without requiring any manual intervention on an individual plant basis. This scalability and lack of human interaction will contribute to the method's adaptability for a large scale phenotypic study regulated by genotypes, and also the method can be applied in the study of quantitative genetic engineering to identify loci controlling variation in the 3D phenotypic traits.

We also introduce a comprehensive taxonomy of 3D phenotypes, first of its kind, that addresses some of the pitfalls associated with 2D single view images due to self-occlusions and leaf crossovers. In the proposed study, either the plant is considered as a single object or as a composite of its components to compute holistic and component structural phenotypes respectively, from the voxel-grid of the plant. The study focuses on maize plants as this species along with rice and wheat, directly or indirectly provide over half of the total world caloric consumption each year. 3DPhenoMV introduces a voxel overlapping consistency check followed by point cloud clustering technique to detect and isolate individual leaves and stem of the maize plants to compute the component phenotypes. To evaluate the proposed method, we also provide a publicly available benchmark dataset called the University of Nebraska-Lincoln 3D Plant Phenotyping Dataset (UNL-3DPPD). The dataset will foster research in 3D image-based plant phenotyping, facilitate new algorithm development, and enable uniform comparisons among competing methods.

## 2. Method

3DPhenoMV consists of four key modules: (a) camera calibration; (b) 3D plant voxel-grid construction; (c) plant component detection and separation; and (d) computation of holistic and component phenotypes.

**Figure 1:**
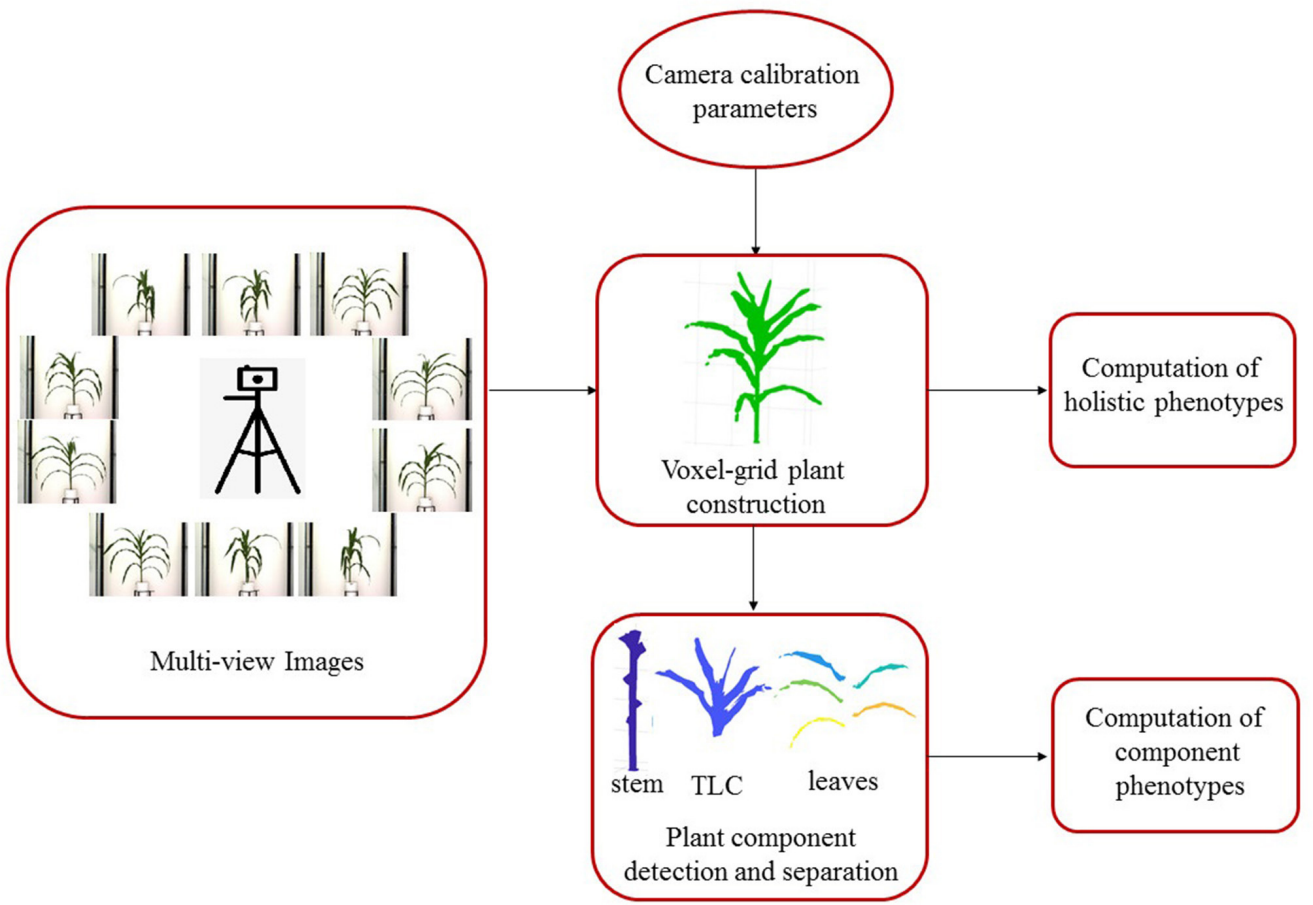
The block diagram of the proposed method.

Figure 1 shows the block diagram of 3DPhenoMV. The images of a plant captured from multiple side views are used as the input to the algorithm. Before the 3D volumetric representation of the plant is reconstructed, the camera parameters must be computed in a calibration step. The 3D model of the plant is reconstructed using the multiple side view images and camera parameters. A set of 3D holistic phenotypes are computed from the 3D model. The individual components of the plant are detected and separated to compute the 3D component phenotypes. These modules are discussed in detail next.

### 2.1. Camera Calibration

**Figure 2:**
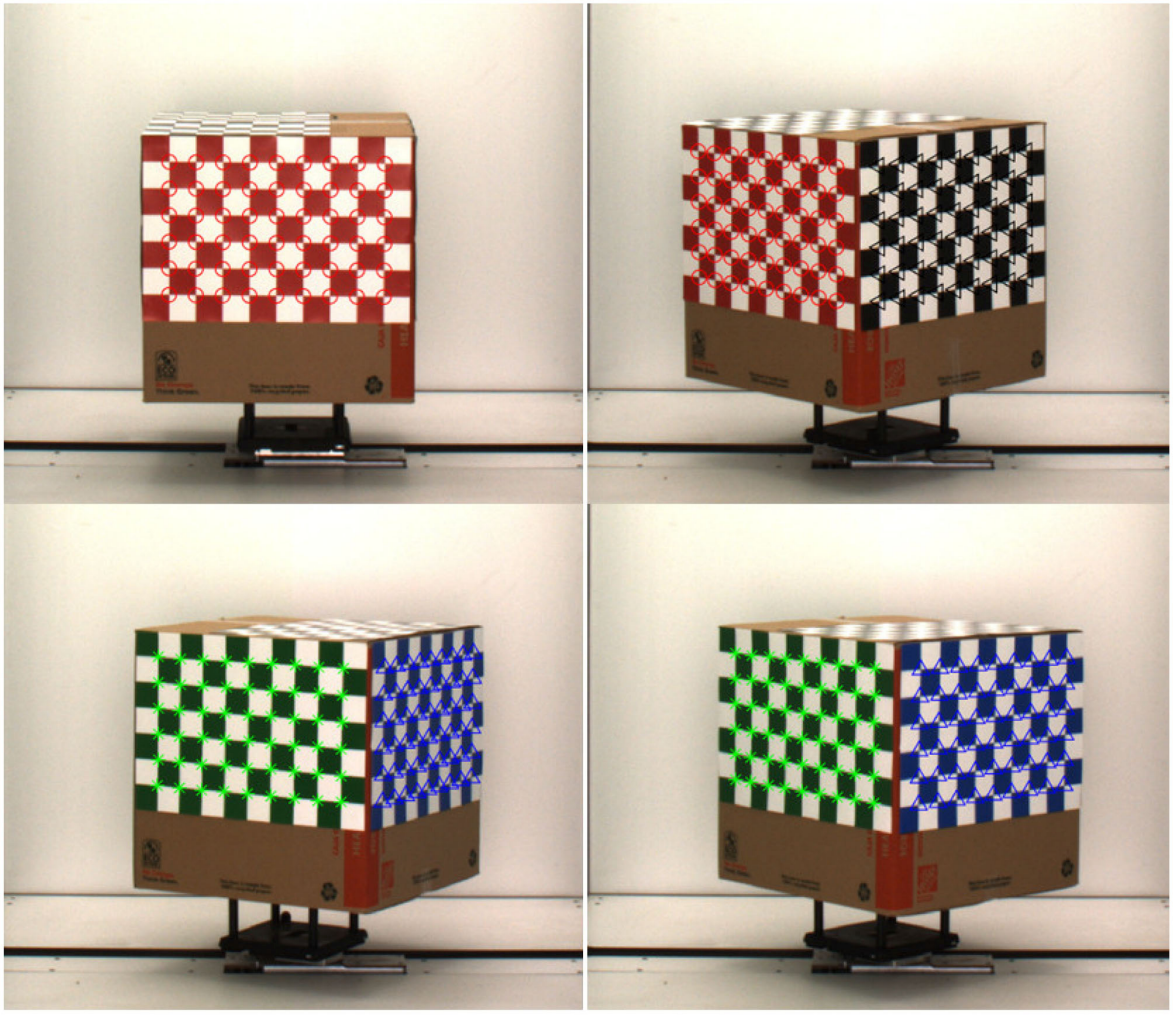
Camera calibration process: checkerboard images with detected corners.

Camera calibration is an essential prerequisite for accurate reconstruction of 3D models based on projective geometry (Ji and Zhang, 2001). It is also necessary to account for geometrical lens distortion. The process involves the determination of intrinsic (e.g., focal length, skew and optical center) and extrinsic (e.g., location and rotation of the camera in space) camera parameters to relate the 2D image pixel coordinates to object points in a 3D reference coordinate system. A cube with checkerboard patterns on side surfaces is used as the calibration target (see Figure 2). The cube is then fitted to the metallic and composite carrier placed on the movable conveyor belt of the HTP3, which moves the calibration cube to the imaging chamber for capturing images in the visible range from multiple side views.

The calibration algorithm described in Heikkila and Silven (1997) involves direct linear transformation based on the collinearity principle of pinhole camera model, where each point in the object space is projected by a straight line through the projection center into the image plane. First, a set of images of the calibration target is derived from multiple viewing angles. Then, interest points from the images are identified, aligned, and the camera parameters are obtained by simultaneous estimation of the parameters based on the Levenberg-Marquardt optimization method. We captured 40 images of the checkerboard cube at 9° intervals. The calibration algorithm uses Harris corner detection algorithm to detect the corners of the 2D checkerboard images. It is evident from Figure 2 that each corner of all four checkerboard surfaces are correctly detected. The additional steps to compensate for radial and tangential distortions due to circular features add robustness to the method. We used camera calibrator app of image processing and computer vision toolbox of Matlab based on this algorithm to compute the camera parameters. To determine the orientation of the checkerboard pattern, it is important that the checkerboard pattern should not be a square, i.e., one side must contain an even number of squares and the other side an odd number of squares. The measurement of the square is required for calibration. The dimension of each square of our checkerboard pattern is 2.5 × 2.5 inches.

### 2.2. 3D Plant Voxel-Grid Construction

**Figure 3:**
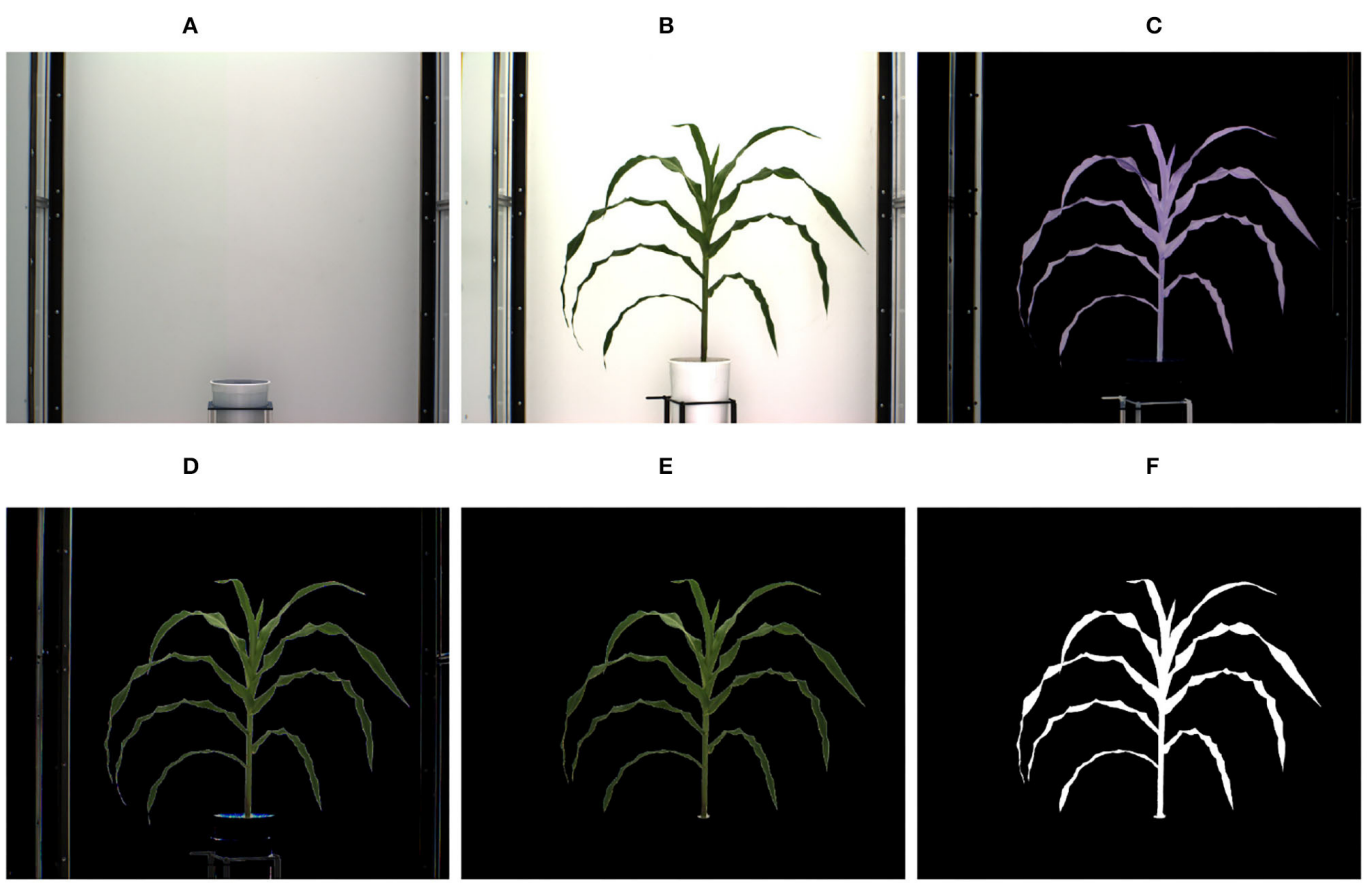
Illustration of segmentation process: **(A)** background image; **(B)** original image; **(C)** foreground obtained after applying frame differencing technique; **(D)** foreground obtained by green pixel superimposition; **(E)** foreground containing green pixels characterizing the plant; and **(F)** binary image.

A voxel is a unit of graphic information that defines a value of a regular grid in 3D space. The first step for 3D model reconstruction of a plant as a grid of voxels (referred to as 3D plant voxel-grid construction) is to segment the plant (foreground), from the background, i.e., the part of the scene which remains static over the period of interest, from images of the plant captured from multiple views. Since, the imaging chambers of HTP3 have a fixed homogeneous background, the simplest background subtraction technique based on frame differencing is often adequate to extract the plant from the background. However, different segmentation techniques may be required in more complex imaging setups. The successful execution of the frame differencing technique requires the background and foreground images to be aligned with respect to scale and rotation. Hence, prior to applying frame differencing technique of background subtraction, we used automated image registration technique based on feature matching to account for change in zoom levels (resulting in scale variation) during the image capturing process. Figures 3A,B show the background image without any plant and an image of a sample plant, respectively.

The extracted foreground obtained using the frame differencing technique is shown in Figure 3C. This image retains some pixels corresponding to the background due to lighting variations, and also some undesirable parts, e.g., soil, soil covering film, etc. In order to remove resulting noises due to variation in lighting, the green pixels of the original image are superimposed onto Figure 3C, which results in the image as shown in Figure 3D. The green pixels constituting the plant are retained, while nosy pixels of other colors are set to zero values to make them part of the background. Thus, the noises are removed. The resulting foreground consisting of only green pixels characterizing the plant is shown in Figure 3E. A color-based thresholding in HSV (Hue, Saturation and Value) color space is applied on this image. The thresholds will depend on the imaging environment, camera characteristics and the reflectance properties of the plant, and can be empirically determined. The following ranges were found to be effective for our imaging setup: hue (range: 0.051–0.503), saturation (range: 0.102–0.804), and value (range: 0.000–0.786). The resulting binary image is subjected to connected-component analysis involving morphological erosion to remove noisy pixels and dilation to fill up any small holes inside the plant image to derive a single connected region as shown in Figure 3F.

**Figure 4:**
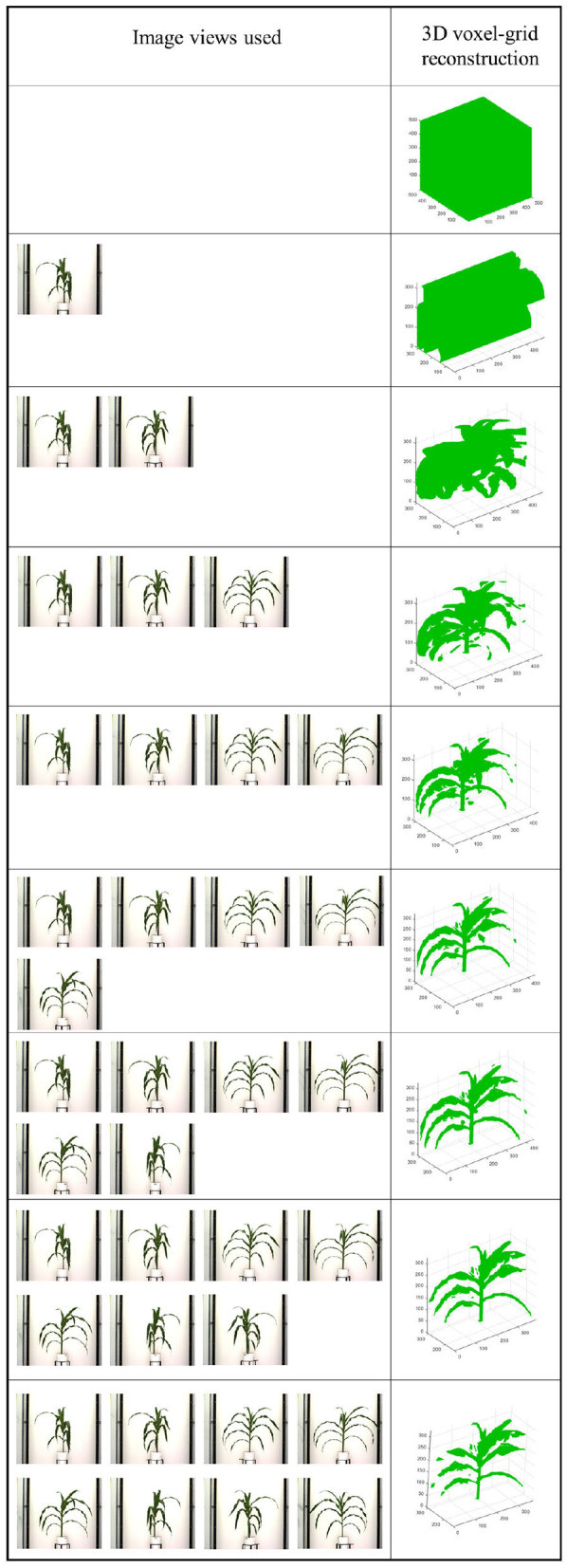
Illustration of 3D plant voxel-grid reconstruction using iterative space carving algorithm, where the right cell shows the reconstructed plants using the image views in the left cell for each row.

After the silhouettes (binary images) of the plant from multiple views are obtained, the 3D model is reconstructed using a space carving approach extended by photo-consistency theory, as explained in Kutulakos and Seitz (2000). Given an initial volume that contains the scene, the algorithm proceeds by iteratively removing (i.e., “carving”) portions of that volume until it converges to the photo hull which is defined as the maximal shape that encloses the set of all photo-consistent silhouettes (Kutulakos and Seitz, 2000). Figure 4 shows the 3D voxel-grid reconstruction of a plant iteratively using the space carving approach. The images in the right cell show the reconstructed 3D plant using the side view images shown in the corresponding left cell for each row. The figure shows that the accuracy in the voxel-grid reconstruction increases with the number of views.

### 2.3. Plant Component Detection and Separation

**Figure 5:**
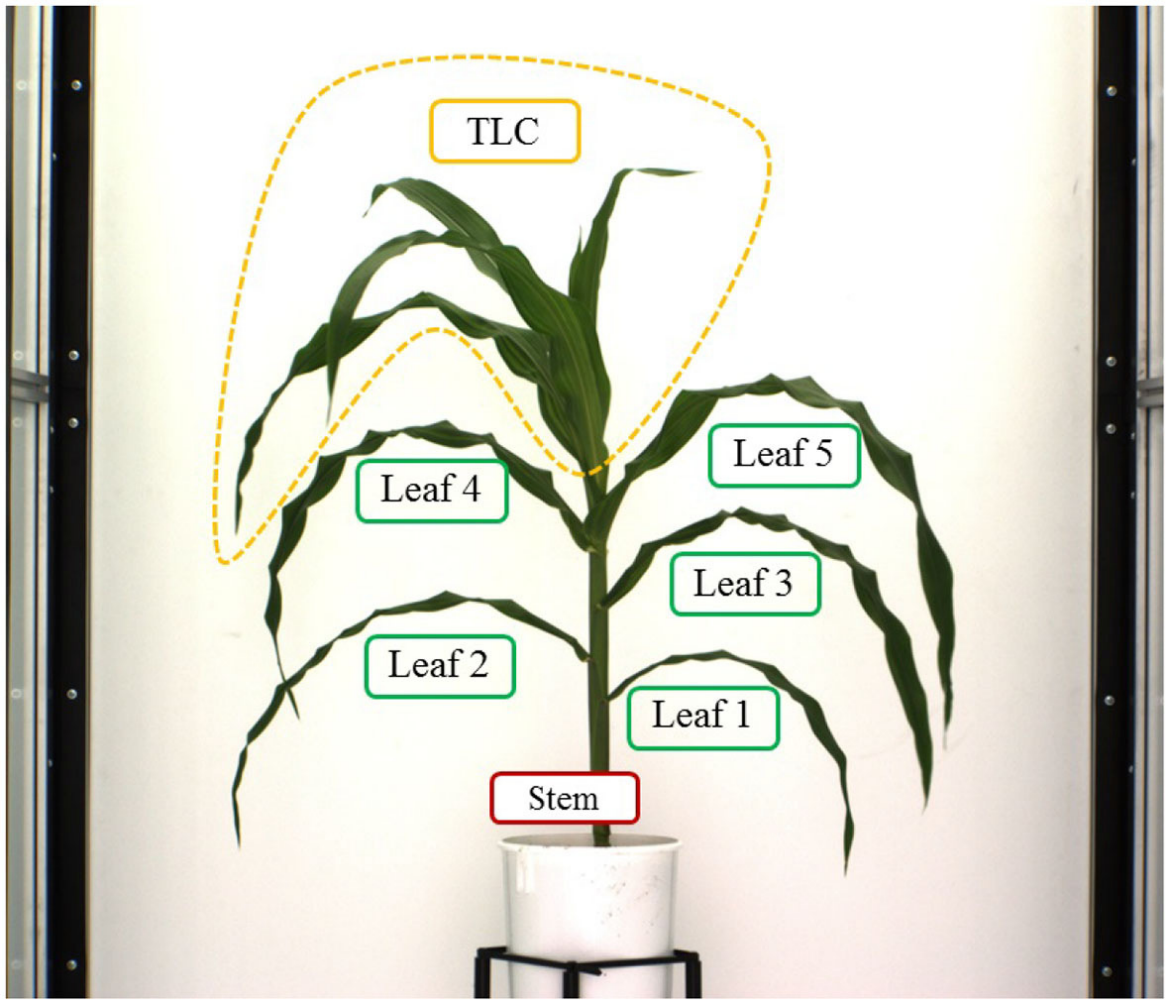
Illustration of plant component labeling. ‘Key’: TLC, top leaf cluster.

Here, we explain the overlapping consistency check and point cloud clustering techniques to divide the 3D plant voxel-grid into three components based on the structure of the plants: stem, leaves and top leaf cluster (TLC) to compute component phenotypes. The TLC denotes the top part of the plant where the last few newly emerged leaves are not attached to distinct junctions, instead, multiple incompletely unfolded leaves surround a single junction occluding each other. In this paper, we are interested in computing phenotypes of full-grown leaves. The endpoint of the stem is considered as the location from where the TLC emerges. These components, i.e., stem, each full-grown leaf and the TLC are shown in Figure 5. The steps to separate these components from the voxel-grid are summarized below.

- Stem: The height of the bounding cube enclosing the reconstructed plant voxel-grid (*VG*) is considered as the height of the plant. The cross-section of VG at height = 0 denotes the base of the stem. We then proceed along height and consider the next adjacent cross-section at height = 1. At this level, we count the total number of cross-sections, and measure their areas by the number of constituent voxels. if the number of cross-section at the next level is still one, we conclude that the cross-section is part of the stem and add it to the stem. if the number of cross-section is two, then we conclude the presence of a junction with a leaf. Note that this assumption holds true for maize plants, as in maize plants leaves emerge using bottom-up approach in alternate opposite fashion, and a single leaf is attached to each junction. We then compute the area of overlapping regions of each of these two cross-sections with the cross-section at the previous level. The cross-section which shows the maximum overlapping with the previous level based on the number of voxels, is considered to be the most consistent cross-section with the previous one, and is considered as the part of the stem. This technique is called as the voxel overlapping consistency check. At this point, we form a frustum of a right circular cone with the two adjacent cross-sections of the stem as the two parallel surfaces of the frustum. All voxels not included in this frustum are discarded. This process is called stem trimming. The iterative process continues until it reaches the TLC, i.e., the termination condition. When TLC is reached, there is no one or two distinct cross-sections, instead a group of several cross-sections are formed with much higher area. The part of the plant thus extracted, is the stem of the plant.
- Top leaf cluster (TLC): The part of the plant enclosed between the endpoint of the stem and the upper horizontal side of the bounding cuboid enclosing the plant is considered as TLC.
- Leaves: Once the stem and TLC are separated out from the plant, the voxels that remain (denoted as leaf point cloud), are the constituents of leaves. We use pcsegdist() function of Matlab R2018a to segment the leaf point cloud into different clusters based on minimum Euclidean distance value of 0.5 between points in different clusters. The points belonging to each cluster represent a leaf. The function pcsegdist() assigns an integer cluster label to each point in the point cloud, and returns the labels of all points. The total number of clusters returned as the output of the function represents the total number of leaves.

### 2.4. Phenotype Computation

**Figure 6:**
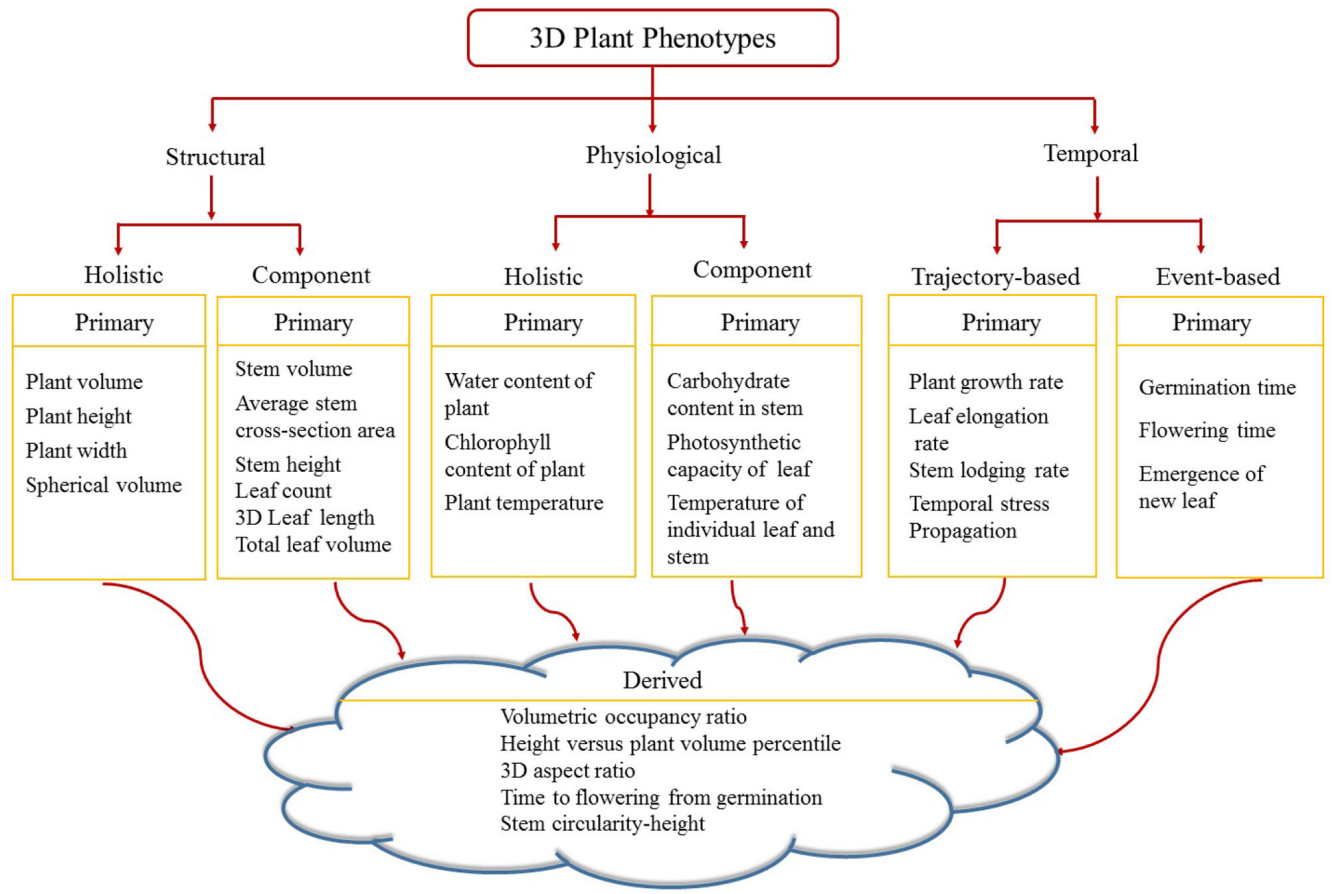
A taxonomy of 3D plant phenotypes.

Figure 6 shows the proposed taxonomy of 3D phenotypes. Image-based above-ground 3D plant phenotypes are broadly classified into three categories, namely structural, physiological and temporal. Structural phenotypes are based on morphological characteristics of the plants, whereas physiological phenotypes refer to the physiological processes in plants regulating growth and metabolism. Structural and physiological phenotypes are grouped into two categories: holistic and component. Holistic phenotypes consider the whole plant as a single object to compute basic geometric properties, e.g., the volume of convex-hull enclosing the plant to account for the size of the plant. The component phenotypes consider the individual parts of a plant, e.g., leaf, stem, flower, and fruit. Examples of 3D component phenotypes include stem volume, cross-sectional area of the stem, total number of leaves, leaf curvature, leaf thickness, flower and fruit volume. Temporal phenotypes are either trajectory-based to account for variations of the static phenotypes with respect to time, e.g., plant growth rate, or event-based which records the important events in a plant's life cycle, e.g., germination and emergence of a new leaf. If a phenotype is based on a single attribute, it is called primary phenotype, e.g., stem height, leaf length and flowering time. A derived phenotype combines two or more primary phenotypes, e.g., the ratio of stem circularity to plant height, time to flowering from germination, etc. In this paper, we focus on holistic and component structural phenotypes and trajectory-based temporal phenotypes of maize plants.

#### 2.4.1. Holistic Phenotypes

Once the 3D volumetric representation of the plant is constructed, a number of holistic phenotypes can be computed. We list the most significant holistic phenotypes below.

*Plant volume:* It is the number of voxels in the reconstructed voxel-grid of the plant.

*Volumetric occupancy ratio:* It is defined as the ratio of number of plant voxels in the reconstructed 3D plant model to the volume of the 3D convex-hull.

*Spherical volume:* It is measured as the volume of the minimum enclosing sphere of the plant.

*3D aspect ratio:* It is defined as the ratio of the height (vertical axis) of the bounding cube of the plant to either the width (longer horizontal axis) or the depth (shorter horizontal axis) of the bounding cube. Based on these two choices, we define two types of aspect ratios: primary and secondary. The primary and secondary aspect ratios are defined as

(1) $$\begin{array}{r} PAR_{p}=\frac{Height_{BC}\text{at side view}}{Width_{BC}\text{at side view}}, \end{array}$$

and

(2) $$\begin{array}{r} PAR_{s}=\frac{Height_{BC}\text{at side view}}{Depth_{BC}\text{at side view}}. \end{array}$$

Where, *Height*_*BC*_ denotes the height of the bounding cube, *Width*_*BC*_ denotes the width of the bounding cube and *Depth*_*BC*_ denotes the depth of the bounding cube of the plant in the side views.

#### 2.4.2. Component Phenotypes

We define the following component phenotypes that can be computed after the individual leaves, stem, and the TLC of the plants have been separated. These component phenotypes significantly contribute to the assessment of the plant's vigor.

*Leaf count:* This is the total number of leaves in the plant, and can be computed as the number of clusters in the leaf point cloud.

*3D leaf length:* It measures the length of each leaf. First, the skeleton of the point cloud of each separated leaf is computed using the Laplacian based contraction method as explained in Cao et al. (2010). Then an *n*-th order polynomial, *p*(*x*), is used to approximate the leaf skeleton using polynomial curve fitting based on a least square error approach. It is given by

(3) $$\begin{array}{r} y=p\left(x\right)=p_{1}x^{n}+p_{2}x^{n-1}+p_{3}x^{n-2}+\ldots +p_{n}x+p_{n+1}, \end{array}$$

where, *p*_1_, *p*_1_, …, *p*_*n*+1_ are the coefficients of the best fit polynomial for the leaf skeleton optimizing the least square error. The leaf length is computed as

(4) $$\begin{array}{r} \int_{x_{1}}^{x_{2}}\sqrt{1+{\left(dy/dx\right)}^{2}}, \end{array}$$

where, *x*_1_ and *x*_2_ denote the *x*-co-ordinates of the two extreme points of the skeleton.

*Total leaf volume:* It is measured by the number of voxels in the leaf cluster.

*TLC volume:* It is measured by the number of voxels in the TLC.

*Stem height:* Given stem *S* = {(*x*_*i*_, *y*_*i*_, *z*_*i*_)}, the height of the stem (*S*_*h*_) is computed by

(5) $$\begin{array}{r} S_{h}=z_{max}-z_{min},\;\text{where,}\\ z_{max}=z:\forall \left(x_{i},y_{i},z_{i}\right)\in S,z\geq z_{i}\\ z_{min}=z:\forall \left(x_{i},y_{i},z_{i}\right)\in S,z\leq z_{i} \end{array}$$

*Stem volume:* It is measured by the number of voxels in the stem.

*Average stem cross-section area:* Stem cross-section area is measured as the total number of voxels in the transverse plane of the stem at any stem height. The average cross-section area over the height of the stem is a useful phenotype.

*Stem circularity-height:* The rate of change of stem cross-section area with respect to stem height is introduced as stem circularity-height phenotype. Stem circularity-height is an example of a trajectory based derived phenotype.

Let us consider a 3D voxel-grid of a plant *P* of size *M* × *N* × *H* be aligned with *X*, *Y*, *Z* axes, respectively. *H* is the height of the plant, which is computed as the height of the enclosing bounding cube of the plant. Without loss of generality, we assume that the stem of the plant, although not perfectly vertical, is generally aligned with the z-axis. Hence, *P* can be represented by

(6) $$\begin{array}{r} P=\left\{\left(x,y,z\right):VG\left[x,y,z\right]=1\right\}. \end{array}$$

A cross-section, *CS* of the stem of a plant *P*, is a slice obtained at a given height, *h*, and is give by

(7) $$\begin{array}{r} CS\left(P,h\right)=\left\{\left(x,y,z\right):\left(x,y,z\right)\in P\wedge \left(z=h\right)\right\}. \end{array}$$

3DPhenoMV is summarized in Algorithm 1.

**Algorithm 1:** **3DPhenoMV**: Performs voxel-grid construction of a plant from 2D multiview images to compute 3D holistic and component phenotypes.

	Input: The 2D multiview images of a plant, i.e., *MV*= {*v*_1_, *v*_2_,…,*v*_*p*_}, where, *v*_*i*_ denotes the *i*-th view of the image of the plant, ∀ *i* = 1 ≤ *i* ≤ *p*, where denotes the total number of available image views.
	Output: [*H*, *C*]. *H* is a set of holistic phenotypes, i.e., *H* = {*h*_1_, *h*_2_,…, *h*_*n*_}, where *n* is the total number of holistic phenotypes. *C* is a set of component phenotypes, i.e., *C* = {*c*_1_, *c*_2_,…, *c*_*m*_}, where *m* is the total number of component phenotypes.
	*B* = ∅; //The set of silhouettes
1:	**for** *i* = 1:*p* **do**
	*s*_*i*_ = segment(*v*_*i*_); // Segment the image *v*_*i*_ using frame differencing
	*b*_*i*_ = binarize(*s*_*i*_); // Get the binary image
	*B* = *B* ∪ {*b*_*i*_}; // Add the image to the set of silhouettes
2:	**end for**
	VG = space-carving(*B*); // Construct the voxel-grid of the plant
	[*P*, *S*, *T*, *L*] = componentSeparate3D(VG); //Divide plant into three components: stem, leaves and TLC
	MBS = computeMinBoundingSph(*P*); // Compute minimum bounding sphere
	BC = computeBoundingCuboid(*P*); // Compute bounding cuboid
	CV = compute3DConvexhull(*P*); // Compute 3D convex-hull
	MBC = computeMinBoundingCuboid(*P*); //Compute minimum bounding cuboid
	*H* = computeHolisticPhenotypes(MBS, BC, CV, MBC);
	*C* = *C* ∪ computeStemPhenotypes(*S*); //Compute stem phenotypes
	*C* = *C* ∪ computeTLCPhenotypes(*T*);// Compute TLC phenotypes
3:	**for** *l*_*i*_ ∈ *L* **do**
	{
	*C* = *C* ∪ computeLeafPhenotypes(*l*_*i*_);//Compute phenotypes for each leaf
	}
	return[*H*, *C*];
	}
4:	**end for**

The algorithm for plant component detection and separation is summarized in Algorithm 2.

**Algorithm 2:** **componentSeparate3D()**: The function performs plant component detection and separation, i.e., divides a plant into three components, i.e., stem, leaves and TLC.

	Input: VG = A voxel-grid derived from a set of views of a plant using space carving.
	Output: [*P*,*S*,*T*,*L*] where
	*P*: 3D point cloud of the whole plant.
	*S*: 3D point cloud of the stem.
	*T*: 3D point cloud of the TLC.
	*L*: 3D point cloud of the leaves.
	componentSeparate3D(VG)
	{
	*P*={(*x*,*y*,*z*):VG[*x*,*y*,*z*]=1}
	*z*_*base*_ = *z*_*min*_: *z*_*min*_ < *z*_*i*_ ∀ (*x*_*i*_, *y*_*i*_, *z*_*i*_) ∈ *P*; //*z* coordinate of plant's base
	*z* = *z*_*base*_;
	done = false; // Set to true when TLC is reached
	while (!done){
	*CS*_*z*_ = CS(*P*, *z*) // Cross-section at *h* = *z*
	*CC*_*z*_ = countCross-section(*CS*_*z*_) //Count number of cross-sections components
	if (|*CC*_*z*_| == 1)
	{
	*S* = *S* ∪ *CS*_*z*_; // Add the voxels to the stem
	}
	else if (|*CC*_*z*_| > 1)
	{
	*CS*_*c*_ = checkComponentConsistency(*CC*_*z*_);// Find the most consistent cross-section
	*CS*_*c*_*t* = trim(*CS*_*c*_); // Trim it to match the previous cross-section
	*S* = *S* ∪ *CS*_*c*_*t*;
	}
	else
	{ // Reached TLC
	*z*_*t*_ = *z*; // *z* coordinate of the bottom of the TLC
	done = true;
	}
	*z*++;
	}
	*T* = {(*x*, *y*, *z*): (*x*, *y*, *z*) ∈ *P* ∧ *z* ≥ *z*_*t*_};// All points above the top of the stem
1:	allLeavesCluster = *P*−*S*−*T*; // Leaves are what is left after removing stem and TLC
2:	*L* = leavesSeparate(allLeavesCluster)// Separate individual leaves using point cloud clustering
	return [*P*, *S*, *T*, *L*];
3:	}

## 3. Benchmark Dataset

### 3.1. Imaging Setup

The Lemnatec 3D Scanalyzer of the high throughput plant phenotyping core facilities at the University of Nebraska-Lincoln (UNL) is used to acquire images for this research. In this system, each plant is placed in a metallic carrier (dimension: 236 × 236 × 142 mm) on a conveyor belt that moves the plants from the greenhouse to the four imaging chambers successively for capturing images in different modalities. The conveyor belt can accommodate up to 672 plants with height up to 2.5 m. It has three watering stations with balance that can add water to target weight or specific volume, and records the specific quantity of water added daily.

The cameras installed in the four imaging chambers are (a) visible light side view and top view, (b) infrared side view and top view, (c) fluorescent side view and top view, and (d) hyperspectral side view and near infrared top view, respectively. Each imaging chamber has a rotating lifter for up to 360 side view images. The specifications of the cameras and detailed descriptions of the HTP3 can be found in Das Choudhury et al. (2018). The average time interval between a plant entering into and exiting from each of the first three imaging chambers for capturing 10 side view images is approximately 1 min 50 s. Since a hyperspectral camera typically captures a scene in hundreds of bands at a narrow interval over a broad range of the spectrum, its image capturing time is significantly higher than that of the other imaging modalities. For our HTP3, the time to capture a single side view image of a plant using a hyperspectral camera (total number of bands: 243; spectrum range: 546–1,700 nm) is approximately 2 min 15 s.

### 3.2. Dataset Description

**Figure 7:**
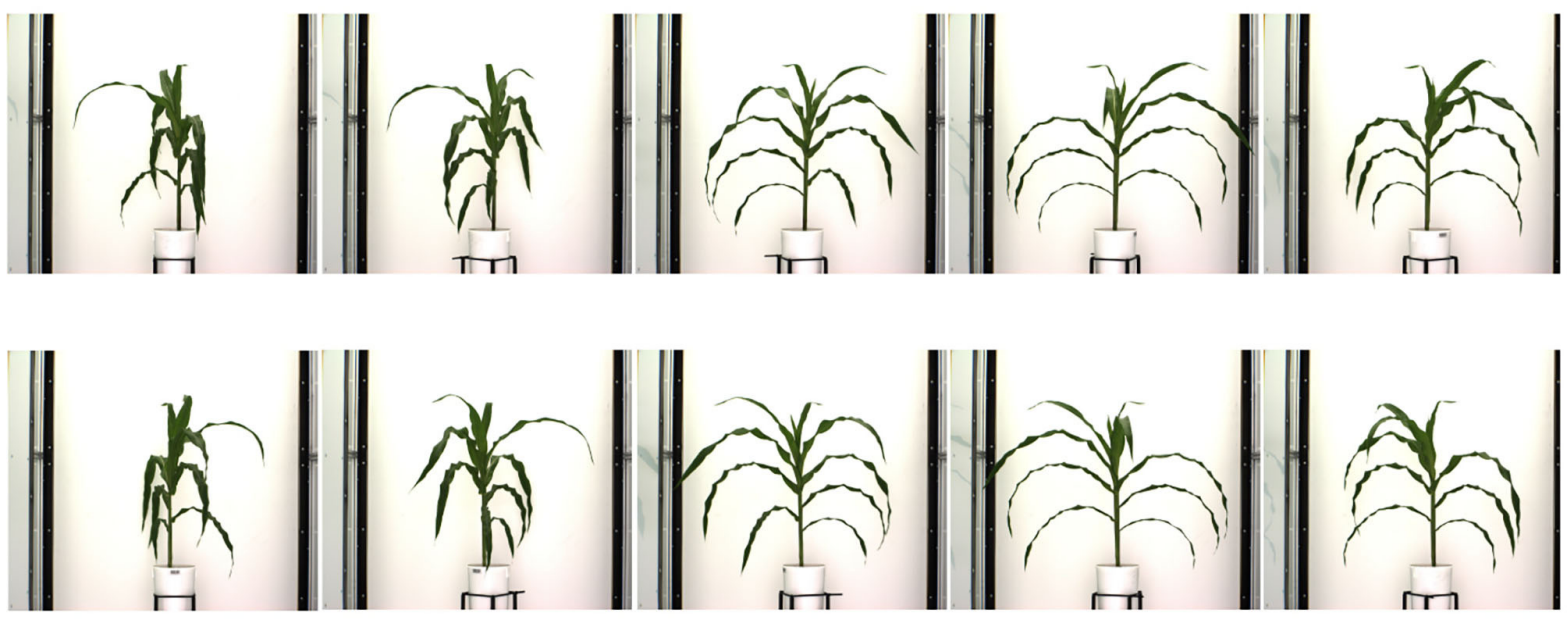
Sample images of a maize plant from UNL-3DPPD dataset for 10 side views.

**Figure 8:**
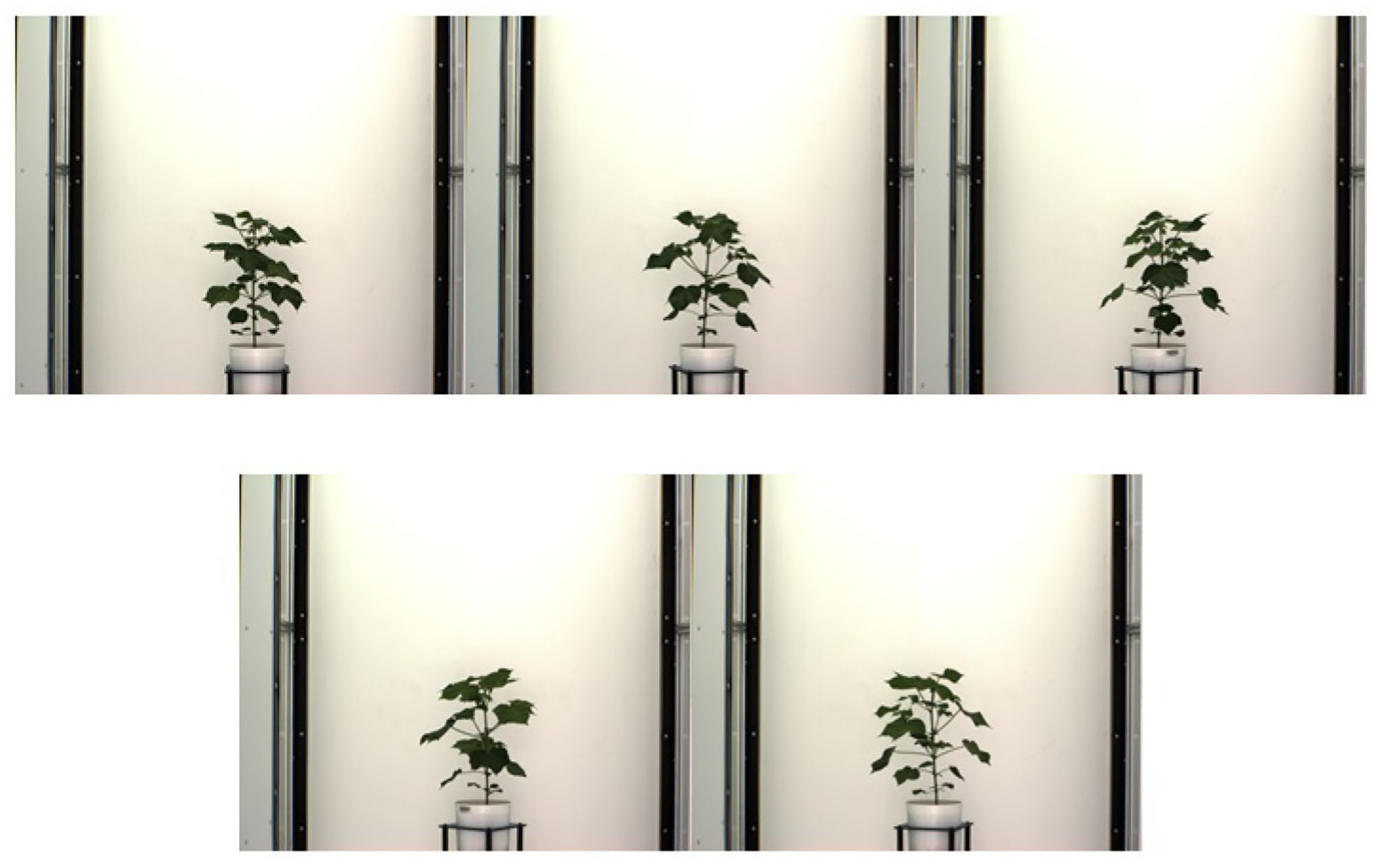
Images of a sample cotton plant captured from five side views.

We introduce a benchmark dataset called UNL-3DPPD to evaluate our algorithms and extraction of holistic and component phenotypic parameters specifically for maize. The dataset is also made publicly available to stimulate 3D phenotyping research, and to encourage researchers to evaluate the accuracy of these algorithms for related crop species with similar plant architectures.^1^ The dataset consists of images of the 20 maize plants for 10 side views, i.e., 0°, 36°, 72°, 108°, 144°, 180°, 216°, 252°, 288°, and 324°. The images were captured daily by the visible light camera for 4 consecutive days. The plants were in their late vegetative stage. Figure 7 shows 10 sample images of a maize plant of the dataset, where each image is captured at 36° intervals of viewing angles. The resolution of each image is 2454 × 2056. Figure 8 shows images of a sample cotton plant captured from five side views, i.e., 0°, 72°, 144°, 216°, and 288°.

## 4. Results

**Figure 9:**
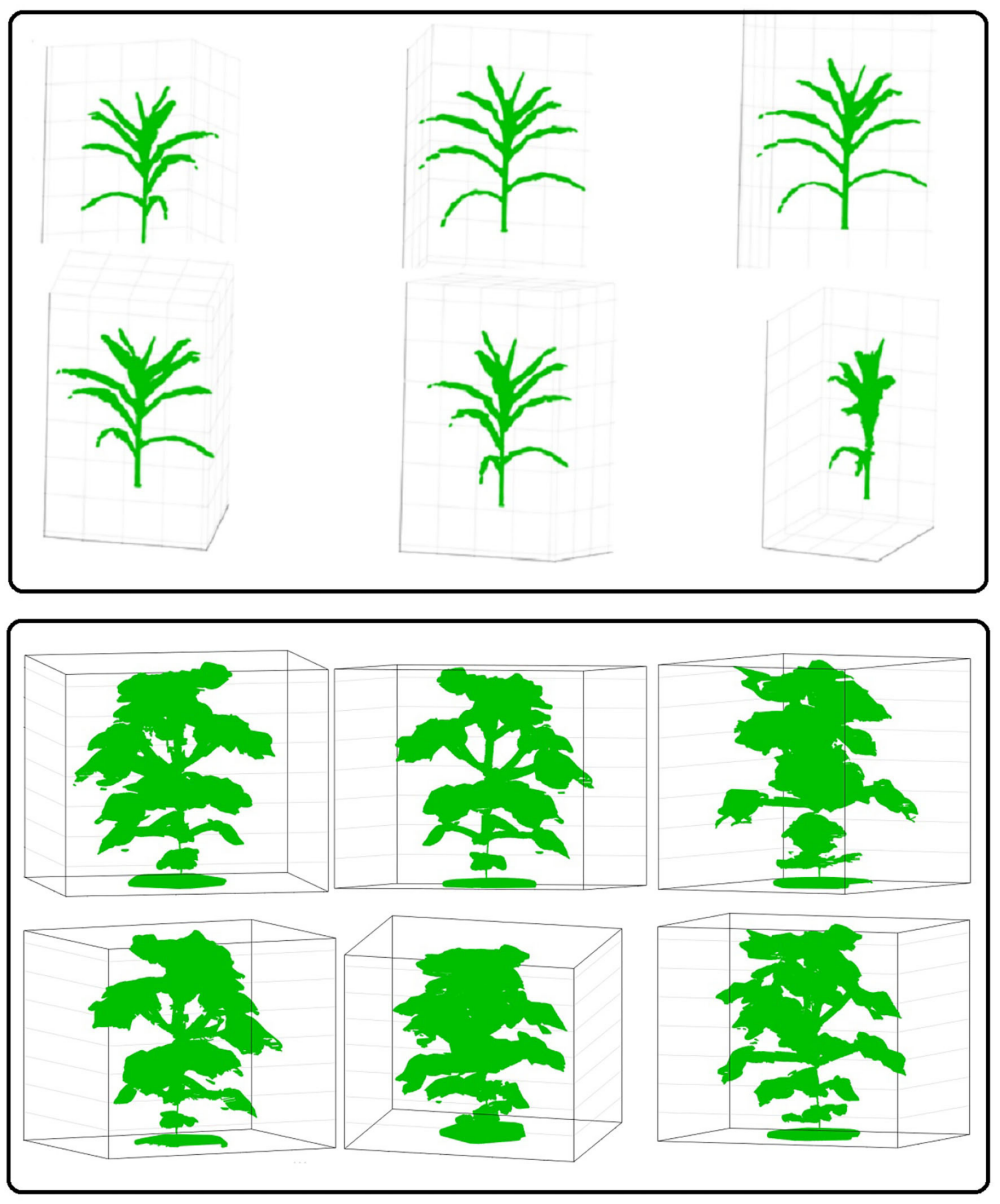
Different views of reconstructed 3D models of plants: rows 1 and 2-maize; and rows 3 and 4-cotton.

We evaluated the performance of 3DPhenoMV algorithm on UNL-3DPPD. Figure 9 (rows 1 and 2) show different views of a 3D plant model reconstructed from 10 side view images of a maize plant. Similarly, Figure 9 (rows 3 and 4) show different views of a reconstructed 3D model of a cotton plant using the five side view images as shown in Figure 8. The experimental results for holistic and component phenotyping analysis, are given below.

### 4.1. Holistic Phenotyping Analysis

**Figure 10:**
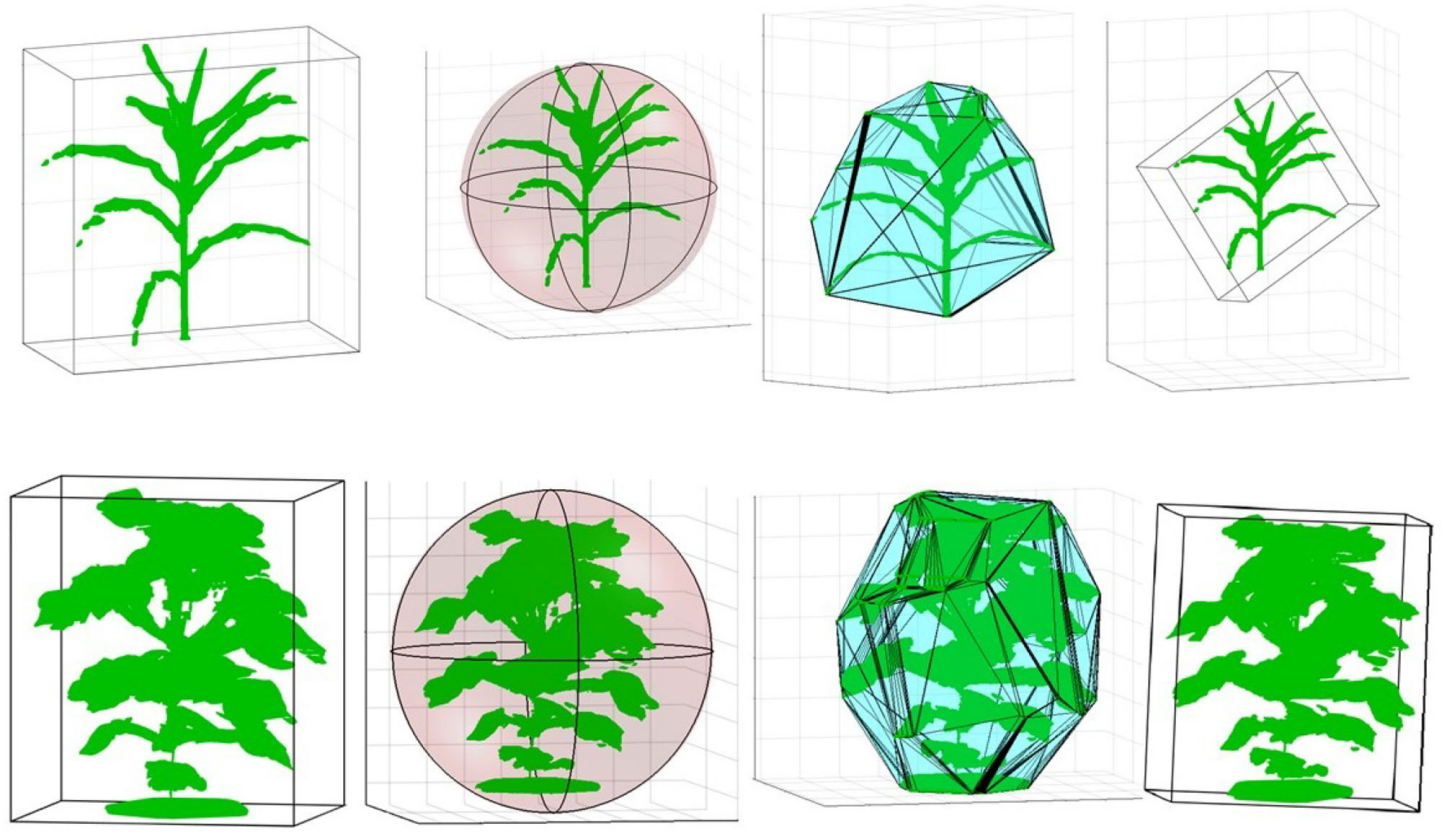
Computation of holistic phenotypes of a sample maize (row 1) and cotton (row 2) plants: bounding rectangular prism; minimum bounding sphere; 3D convex-hull and minimum bounding rectangular prism (left to right).

**Figure 11:**
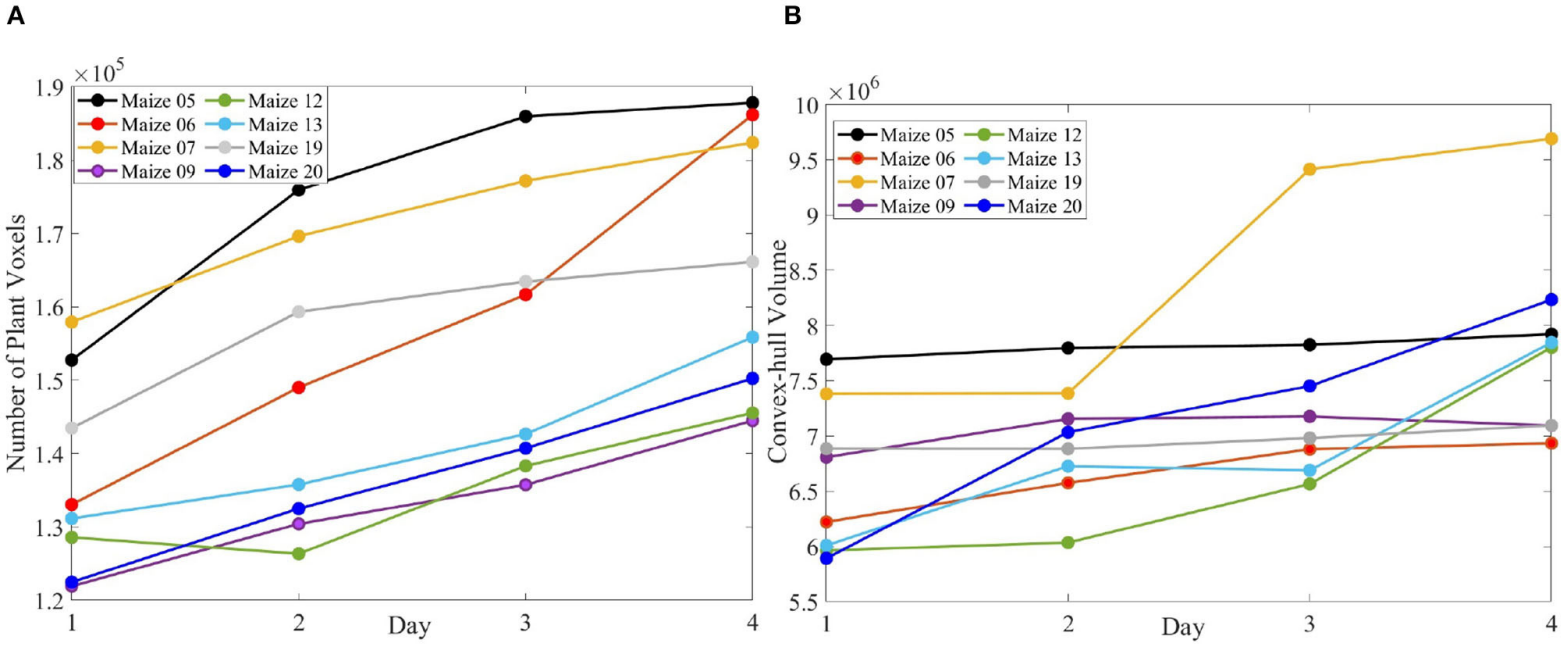
The number of plant voxels of sample maize plants **(A)**; and the volume of convex-hull of the same plants **(B)** over consecutive 4 days.

**Figure 12:**
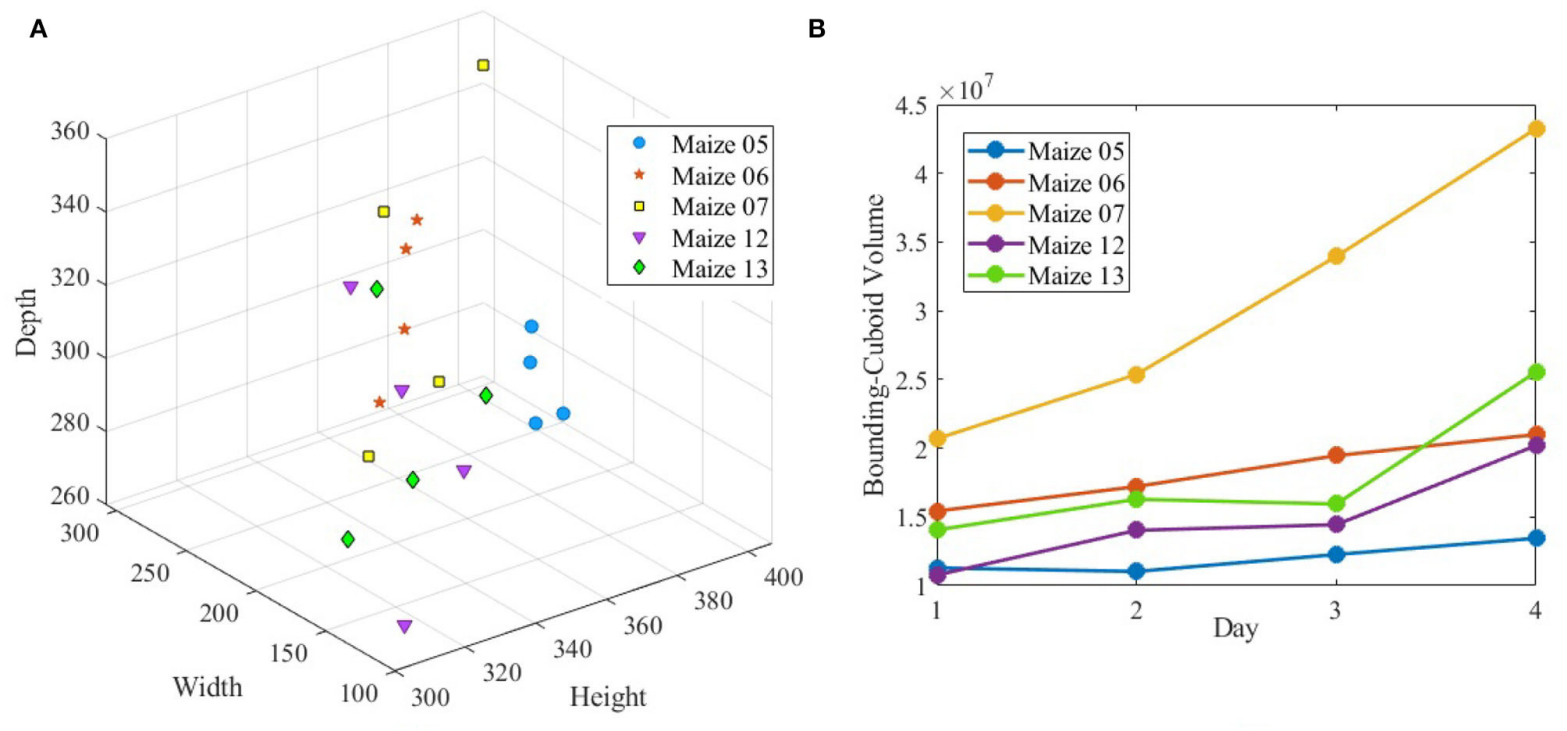
The dimensions of bounding cuboid of sample maize plants **(A)** and the volume of bounding cuboid of the same plants **(B)** over consecutive 4 days.

Figure 10 (row-1) shows a sample 3D volxel grid plant from UNL-3DPPD enclosed by bounding rectangular prism, minimum bounding sphere, 3D convex-hull and minimum bounding rectangular prism. Similarly, Figure 10 (row-2) shows the 3D volxel grid of a sample cotton plant enclosed by bounding rectangular prism, minimum bounding sphere, 3D convex-hull and minimum bounding rectangular prism. Figures 11A,B graphically demonstrate two holistic phenotypes, i.e., the volume of convex-hull and the total number of plant voxels, of several representative plants from UNL-3DPPD for 4 days. The scatter plot in Figure 12A shows the three dimensions of the bounding cuboid enclosing a plant, i.e., height, width and depth, for five maize plants from UNL-3DPPD for 4 consecutive days. The same markers are used to denote the same plant for ease of visualization. Figure 12B shows the trajectories of the bounding volume of the same five maize plants for 4 consecutive days to represent the plant growth rate.

### 4.2. Component Phenotyping Analysis

**Figure 13:**
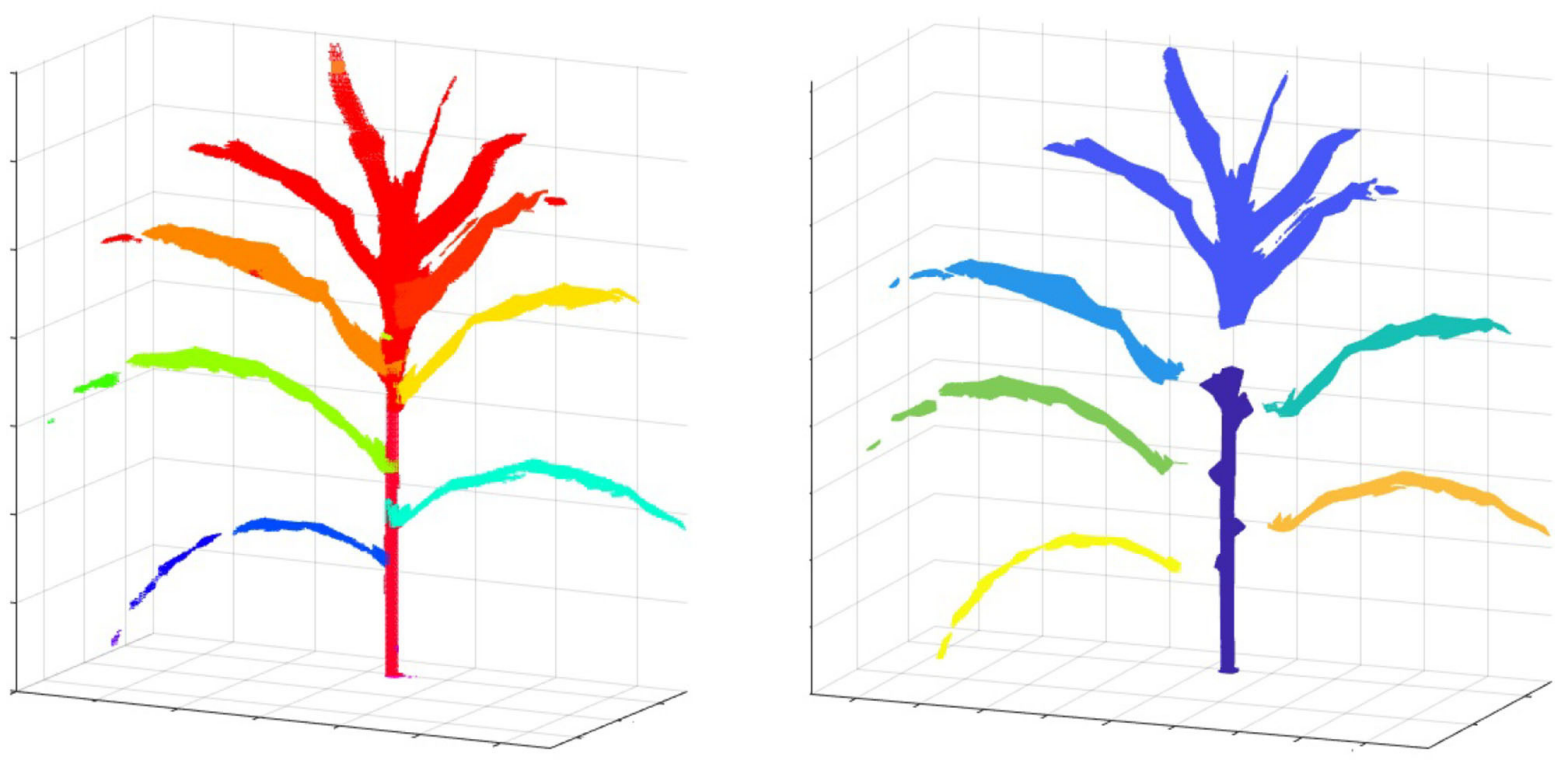
Illustration of plant component segmentation: **(left)** the voxel-grid reconstruction of a plant; and **(right)** plant voxel-grid with its three disjoint components.

To compute the component phenotypes, it is essential to detect and separate individual parts of a plant accurately. The result of plant component detection and separation, i.e., separating full-grown leaves, stem and TLC from the reconstructed plant voxel-grid, is shown in Figure 13. Figure 13 (left) shows the reconstructed voxel-grid of a plant with its three main components, i.e., stem, individual leaves and TLC, shown in different colors. A separated view of these components is shown in Figure 13 (right) for illustration. The figure shows that the total number of full-grown leaves (not included in TLC) are five in number.

**Figure 14:**
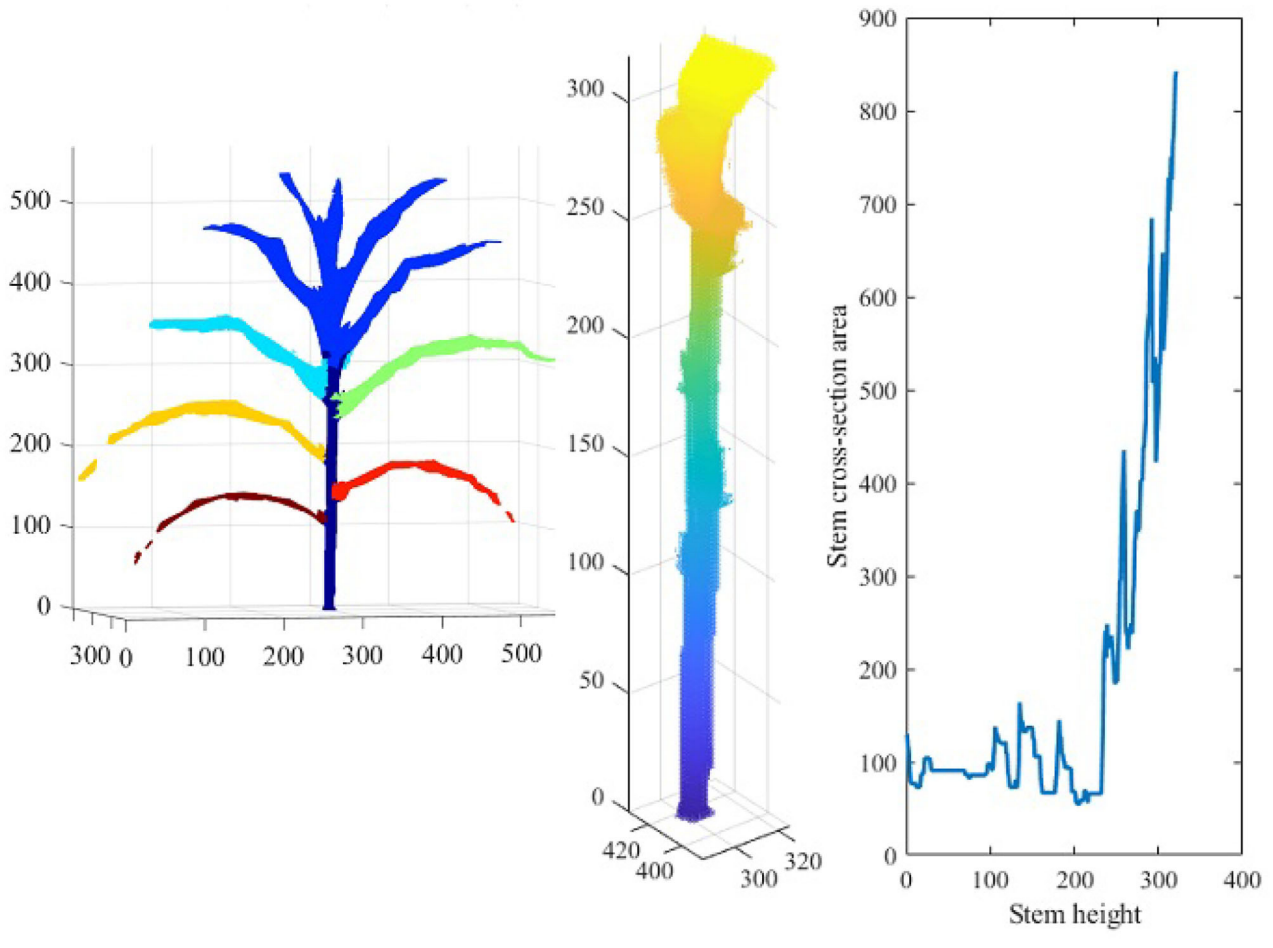
Illustration of stem-based component phenotypes: the 3D voxel-reconstruction of a plant **(left)**; the isolated stem **(middle)**; and line graph showing the rate of change of stem cross-section area as a function of stem height **(right)**.

**Figure 15:**
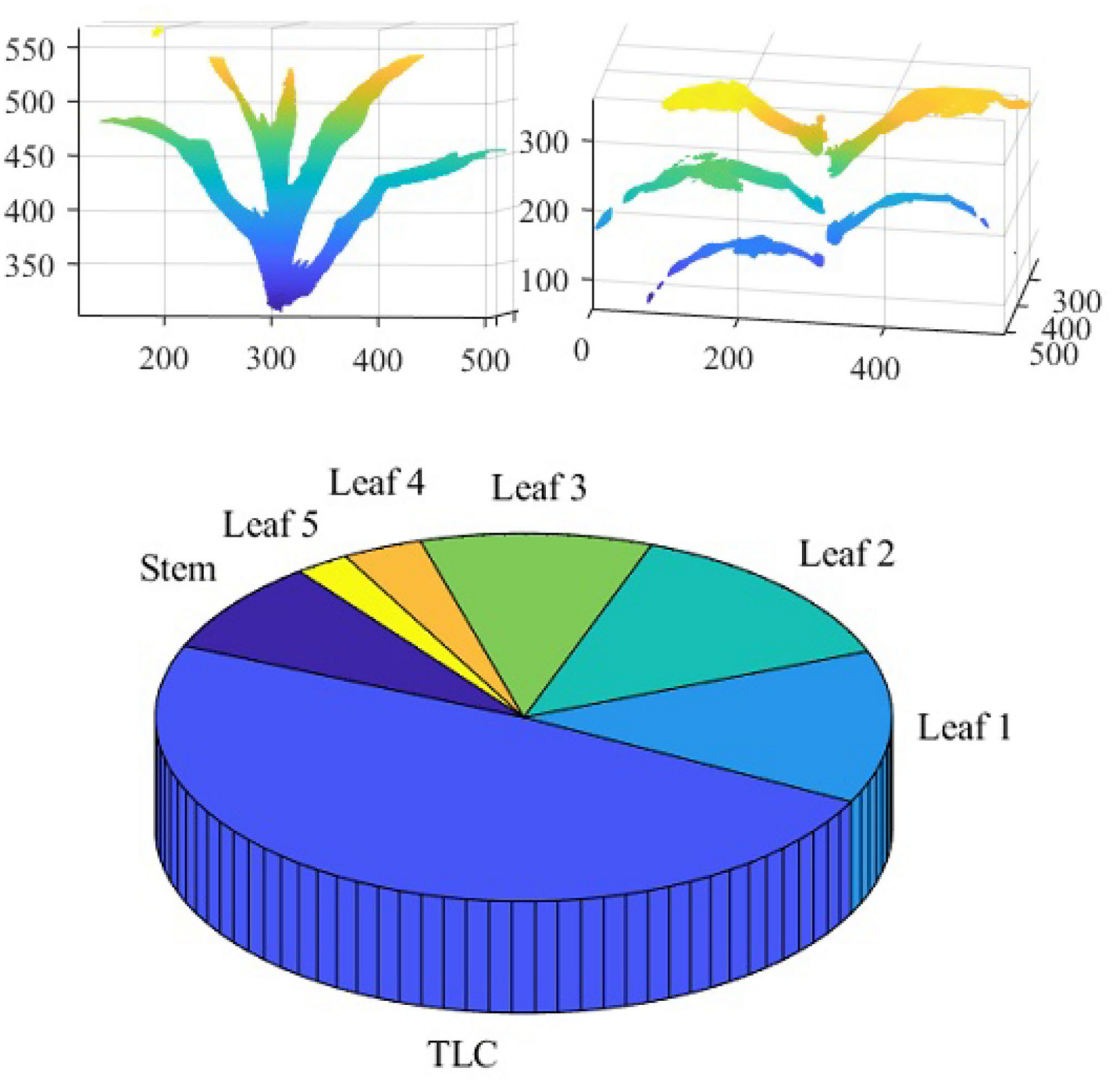
Illustration of leaf-based component phenotypes: isolated TLC **(top left)**; isolated leaves **(top right)** and pie graph showing the total number of voxels constituting stem, TLC and individual leaves **(bottom)**.

Figure 14 shows the reconstructed voxel-grid of a plant (left) and the isolated stem (middle) computed by the plant component detection and separation algorithm. Figure 14 (right) shows the rate of change of stem cross-section area as a function of stem height. Figure 15 shows the isolated TLC and the individual leaves of the plant shown in Figure 14 (left). We also show the volumes of the stem, the TLC and the individual leaves of the plant using a pie chart for comparative analysis. The chart shows that the volume of the TLC is significantly larger than that of the stem, and is comparable to the volume of all the leaves combined. The leaves are numbered in order of emergence. Note that the volume of the leaves are directly related to the order of emergence, e.g., the first leaf has the highest volume, and vice versa.

## 5. Discussion

The number of leaves that emerge in the life cycle of a maize plant are the indicator of the plant's growth stage. The number of leaves present at any point in the plant's life cycle, the size of individual leaves, stem height, rate of growth of stem cross-section area, are the important phenotypes that best represent plant's vigor. However, accurate computation of these phenotypes from 2D projections (images) of 3D plants is not possible in general, especially in the late vegetative growth stages where maize plants exhibit complex architecture due to cluttered leaves. While most state-of-the-art methods have addressed the 3D reconstruction of plants in early growth stages due to simplicity, we contribute in the research advancement of 3D phenotyping analysis of plants in the late vegetative stage by developing an algorithm, introducing a 3D plant phenotyping taxonomy and public release of a multiview benchmark dataset consisting of original image sequences of maize plants and images of checkerboard patterns for camera calibration.

We introduce a novel method, i.e., 3DPhenoMV, to compute 3D holistic and component structural phenotypes based on voxel-grid construction of plants using images captured from multiple viewing angles in a HTP3. Plants like maize are characterized by thin ribbon-like architecture, where some parts of leaves may be as wide as only a few pixels in images. In addition to thin structures, lack of textures in the surface of the plants pose challenges to the accurate 3D reconstruction of plants, often resulting in the disjoint leaf parts (Furukawa and Hernandez, 2013). Unlike maize, cotton plants have a bushy architecture with lots of cluttered leaves around the stem. To demonstrate the efficacy of the 3DPhenoMV to reconstruct the 3D voxel-grid of plants with different architectures, we used five side view RGB images of a cotton plant to reconstruct its 3D model and compute the holistic phenotypes. The creation of a new dataset consisting of images of the different types of plants with varying architectures, e.g., sorghum, tomato, and wheat, and additional view angles, will be examined in the future work.

The significance of the 3D phenotypes computed by 3DPhenoMV are discussed below. Leaves are one of the primary organs of plants which transform solar energy into chemical energy in the form of carbohydrate through photosynthesis, releasing oxygen as a byproduct. Maize plants alter leaf arrangement around the stem (i.e., phyllotaxy) at different stages of the life cycle in response to light signal perceived through the phytochrome to optitmize light interception. The total number, orientation and size of leaves are therefore linked to plant photosynthetic light efficiency and net primary productivity. Thus, the total number of voxels constituting each leaf can be used as a proxy for leaf size, and hence, is an important phenotype. The stem height is a direct measure of a plant's growth. The stem cross-section area is a measurement of toughness of a stem. Stem cross-section area appears to be an important defense mechanism in maize across diverse groups of germplasms. Stem cross-section area as a function of stem height, i.e., circularity-height ratio, provides information on plant's strength, and hence, can be an early signal to lodging susceptibility. Stem lodging is primarily caused due to water logging, nutrient imbalances and deficiencies. Yield loss due to lodging reduces the US corn harvest by 5–25% per year (2.4–12 billion dollars at 2015 corn prices), and hence, a phenotype to measure its susceptibility is important.

The area of convex-hull enclosing the plant computed from 2D image sequences is not an accurate estimation of plant's biomass, because maize plants may rotate for shade avoidance (Das Choudhury et al., 2016). This is evident from the fact that graph for the area of convex-hull as a function of time oscillates randomly instead of following an increasing trend. The volume of bounding cuboid enclosing the plant provides a more accurate estimation of its biomass. 3D Plant aspect ratio is a metric for distinguishing between genotypes with narrow vs. wide leaf extent after controlling for plant height. The volume of bounding cuboid and 3D plant aspect ratio are important phenotypes that are influenced by the genetic environment interaction. They also help in the study of determining plants' response to environmental stresses. The time rate of variation of these phenotypes (i.e., trajectory-based phenotypes) is also demonstrated in this paper. The graph showing the variation of volume of bounding cuboid as a function of time is a trajectory-based temporal phenotype, and is an illustration of the plant's growth rate.

The 3D voxel-grid reconstruction and 3D phenotype computation algorithms are implemented using Microsoft Visual Studio 2017 Edition on an Intel Core i7-7700HQ processor with 16 GB RAM and Nvidia GeForce GTX 1060 with Max-Q Design 6 GB DDR5 memory working at 2.80-GHz. The computer runs a 64 bit Windows 10 operating system. The original input images are re-scaled to the size of 1200 × 1200 prior to the reconstruction. The algorithms use NVIDIA GPU Computing Toolkit 9.0 and OpenCV 3.3, and the implementation is parallelized on GPU cores using Nvidia's CUDA library. The size of the input voxel-grid is 1200 × 1200 × 1200. The time taken for reconstructing the 3D voxel-grid of a single maize plant (that requires carving out 2402365 total number of voxels) using 10 side views captured by the visible light camera is approximately 3.5 min on this platform.

## 6. Conclusion

The paper introduces a novel method called 3DPhenoMV for 3D voxel-grid reconstruction of maize plants based on images captured from multiple views to compute 3D holistic and component phenotypes in the late vegetative stage. It also provides a broad taxonomy of 3D phenotypes, and publicly disseminates a benchmark 3D dataset for the development and uniform comparisons of algorithms to compute phenotypes for the advancement of 3D image-based high-throughput plant phenotyping research. In addition to maize, the efficacy of 3DPhenoMV for 3D voxel-grid reconstruction is demonstrated using a sample cotton plant which assumes more complex architecture than a maize plant, due to the presence of cluttered leaves around the stem. The promising reconstruction performance of cotton using half of the number of side views that are used for maize reconstruction, shows the potential of the method to be extended to other plant architectures.

We use the space carving approach to construct the 3D voxel-grid of a plant using multiple side view images. We compute a set of holistic phenotypes by considering the plant as a whole, e.g., volumetric occupancy ratio to estimate the biomass yield and 3D aspect ratio which provides information on canopy architecture. It is essential to detect and separate the individual components of the plant to compute component phenotypes. Thus, we use point cloud clustering and overlapping consistency check methods to separate individual leaves, stem and the TLC of the plant. A set of component phenotypes, e.g., total leaf-count, length and volume of individual leaves, stem height, stem circularity-height, stem volume and volume of TLC are proposed in this paper. The component detection and separation algorithm is applicable for plants with distinct stems that are above-ground, not highly branched, and characterized by distinct nodes and internodes. Experimental analyses on UNL-3DPPD consisting of images of maize plants shows the efficacy of the proposed method. Future work will consider creation of a new dataset consisting of larger number plants belonging to different genotypes with additional views, and validation of the phenotypes with physical measurements from the plant, destructively if needed, e.g., for biomass. Additional phenotypes and computational methods to compute them will also be explored in our future research. The method will also be evaluated to estimate the temporal variation of phenotypes controlled by genetic factors for heritability analysis.

## Data Availability Statement

The datasets generated for this study are publicly available from https://plantvision.unl.edu/dataset.

## Author Contributions

SD developed and implemented the algorithm, and led experimental analysis, UNL-3DPPD planning and organization, and the manuscript writing. SM developed and implemented the algorithm, and performed experimental analyses. AS supervised the research, provided constructive feedback throughout the algorithm development process and critically reviewed the manuscript. VS facilitated the dataset formation. SM, AS, and TA contributed to the manuscript writing. All authors contributed to the article and approved the submitted version.

## Conflict of Interest

The authors declare that the research was conducted in the absence of any commercial or financial relationships that could be construed as a potential conflict of interest.

## Footnotes

^1^UNL-3DPPD can be freely downloaded from http://plantvision.unl.edu/.
